# Targeting Tn Antigen Suppresses Aberrant O‐Glycosylation‐Elicited Metastasis in Breast Cancer

**DOI:** 10.1111/jcmm.70279

**Published:** 2024-12-09

**Authors:** Tan Du, Xichen Dong, Jingyu Tan, Xiangyu Chen, Jian Liu, Tao Wen, Xiaoli Ru

**Affiliations:** ^1^ Medical Research Center, Beijing Chao‐Yang Hospital Capital Medical University Beijing China; ^2^ Department of Gynecology and Obstetrics, Beijing Chao‐Yang Hospital Capital Medical University Beijing China

**Keywords:** breast cancer, HPA, metastasis, O‐glycosylation, Tn antigen

## Abstract

The Tn antigen, a truncated O‐glycan representing aberrant mucin‐type O‐glycosylation, is frequently observed in human breast cancer. However, the functional role of Tn antigen in breast cancer metastasis remains insufficiently investigated. This study aimed to elucidate the expression profile of Tn antigen in breast cancer and its potential as a therapeutic target for inhibiting metastasis. Immunohistochemical staining was performed to determine the levels of Tn antigen expression in breast cancer tissues and its clinical relevance was analyzed accordingly. Tn‐positive breast cancer cell lines were generated through disruption of the Cosmc gene. The functional roles of Tn antigen in breast cancer metastasis were studied in both in vitro and in vivo models. Western blotting and immunofluorescence staining were employed to investigate the molecular mechanisms by which Tn antigen promotes breast cancer metastasis. Our findings revealed that Tn antigen was prevalent in breast carcinomas, particularly within metastatic lesions. Tn antigen expression was positively correlated with lymph node metastasis and poorer patient survival. Tn antigen‐expressing breast cancer cells exhibited enhanced invasiveness and metastasis, along with significant activation of EMT and FAK signaling pathways. Targeting Tn‐positive cells with HPA (*Helix pomatia agglutinin*) demonstrated the suppression of invasive and metastatic capabilities, EMT program, and FAK signaling in vitro, as well as reduced pulmonary metastasis in a xenotransplant mouse model. This study reveals that Tn antigen‐mediated aberrant O‐glycosylation plays a contributing role in breast cancer metastasis, which may serve as a potential therapeutic target in clinical practice.

## 1 Introduction

Breast cancer has been the most common malignancy and the second leading cause of cancer‐related deaths for women throughout the world [1]. Although early diagnosis and treatment have greatly improved, metastasis remains a significant survival challenge for patients [2, 3]. According to the recent reports, the overall 5‐year survival rate for primary breast cancer patients exceeds 80%; nevertheless, that of patients with distant metastasis is discouragingly less than 25% [4, 5]. The mechanisms underlying breast cancer metastasis are still poorly understood. There is an urgent need to identify the key regulators during the metastatic process, which can be translated to therapeutic targeting.

Altered glycosylation has frequently been detected in a majority of human carcinomas, characterized by abnormal expression of immature glycan structures, such as the Tn antigen (GalNAc‐Ser/Thr) [6]. Tn antigen is a truncated O‐glycan that has been found to express abundantly in many types of human cancers, including breast cancer, and is associated with cancer aggressiveness and poor prognosis, whereas its expression is absent in the normal tissues [7, 8], thereby highlighting Tn antigen as an attractive therapeutic target in cancer treatment. Generally, Tn antigen is normally modified by the enzyme core 1 β1,3‐galactosyltransferase (T‐synthase) to form mature core 1 O‐glycans (Galβ1‐3GalNAcα1‐Ser/Thr, T antigen), which are the most common glycan structures expressed in normal mammary gland epithelium [9]. Of note, T‐synthase activity requires a specific molecular chaperone termed Cosmc, which helps T‐synthase fold correctly in the endoplasmic reticulum [10]. Loss‐of‐function or deficiency in Cosmc results in degradation of T‐synthase, followed by accumulation and exposure of Tn antigen [11]. Although expression of the Tn antigen has been widely detected in most breast carcinomas, its functional impact on cancer metastasis is not well defined.

In the present study we found that Tn antigen was not only prevalently expressed in human breast cancer but also was significantly associated with metastasis, indicating a metastasis‐promoting role for Tn antigen in breast cancer. To address this issue, we forcibly induced expression of Tn antigen in BT549, MDA‐MB‐231 highly metastatic breast cancer cell lines by disruption of Cosmc. Both in vitro and in vivo experiments showed that Tn antigen expression promoted epithelial‐mesenchymal transition (EMT) and enhanced cancer cell metastasis. Moreover, Tn antigen revealed to increase cancer cell protrusion formation, potentially linked to activation of the focal adhesion kinase (FAK) signaling pathway. Masking Tn antigen by HPA (*Helix pomatia agglutinin*), a lectin that can specifically recognize and bind Tn antigen [12, 13], demonstrated to diminish metastasis‐enhancing effects of Tn‐positive cells, suggesting that Tn antigen may represent an promising target for therapeutic intervention against breast cancer metastasis.

## 2 Materials and Methods

### 2.1 Tissue Samples

Human primary and metastatic breast cancer tissues, as well as adjacent normal tissues, were obtained from patients at Beijing Chao‐Yang Hospital, Capital Medical University, Beijing, China. The ethical approval was granted by the Ethics Committees of Beijing Chao‐Yang Hospital, Capital Medical University, in accordance with the Declaration of Helsinki guidelines for biomedical research involving human subjects.

### 2.2 IHC Analysis of Tn Antigen

Breast cancer and adjacent normal tissue samples were subjected to immunohistochemistry (IHC) analysis. The 5 μm‐thick paraffin‐embedded tissue sections were initially deparaffinized, rehydrated, and underwent antigen retrieval, followed by incubation with hydrogen peroxide and blocking solution. Subsequently, the primary antibody, a mouse‐anti‐Tn IgM mAb (20 μg/mL, CA3638, clone 12A8‐C7‐F5, kindly provided by Dr. Tongzhong Ju, Emory University, Atlanta, Georgia, USA), was applied to the tissue sections. Following primary antibody incubation, a secondary antibody, goat antimouse IgM (5 μg/mL, Abcam, ab97230), was applied, and DAB staining was performed for visualization.

### 2.3 Cell Culture

Human breast cancer cell lines MDA‐MB‐231 and BT549 and a human embryonic kidney cell line HEK293T were obtained from the American Type Culture Collection (ATCC). BT549 cells were cultured in RMPI‐1640 medium (Gibco, Carlsbad, CA, USA). MDA‐MB‐231 and HEK293T cells were maintained in DMEM medium (Sigma, Santa Clara, CA, USA). All media were supplemented with 10% FBS (Ausbian, Sydney, Australia) and 1% Penicillin–Streptomycin solution (Gibco). All cell lines were incubated at 37°C in a humidified atmosphere with 5% CO_2_.

### 2.4 Transwell and Wound Healing Assays

Cell migration and invasion abilities were assessed using a Transwell plate (BD Bioscience, 8 μm pore size). After 24 h of starvation, cells (5 × 10^4^) suspended in serum‐free medium were added into the upper chamber of Transwell plate precoated with or without Matrigel (BD Bioscience), while 600 μL complete medium with 10% serum was filled into lower chambers. Following culture for 12–24 h, cells that passed the membranes were fixed with 4% paraformaldehyde and stained with 0.1% crystal violet. The migrated and invasive cells were observed under a microscope.

For the wound healing assay, cells (1 × 10^6^) were seeded on a 6‐well plate with three replicate wells. After the cells' confluence reached approximately 90%, a cell‐free gap was created by scratching a confluent monolayer with a pipette tip. The cells were observed and photographed at 24 and 48 h after wounding. The migration capacity was evaluated using the following migration index: migration index (%) = (gap at 48 h)/(gap at baseline) × 100.

### 2.5 Western Blotting Analysis

The cells were harvested, washed with cold PBS, and subsequently lysed using lysis buffer (RIPA, Beyotime, China). After a 20‐min incubation on ice to facilitate lysis, total proteins were collected, and the protein concentrations were determined using a BCA assay kit (Thermo Fisher, MA, USA). Samples were separated by SDS‐PAGE (12% gel) and transferred onto PVDF membranes (Millipore, MA, USA), then blocked with 8% fat‐free milk for 1 h at room temperature. After washing with TBST, membranes were incubated with primary antibodies at 4°C overnight. Primary antibodies against the following antigens were used: Cosmc (0.4 μg/mL, Santa Cruz, sc‐271829), T‐synthase (0.2 μg/mL, Santa Cruz, sc‐100745), E‐cadherin (1:1000, CST, 14472), ZO‐1 (1:1000, CST, 13663), ZEB‐1 (1:1000, CST, 70512), Vimentin (1:1000, CST, 70512), Snail (1:800, CST, 3879), Slug (1:800, CST, 9585), and GAPDH (1:2000, CST, 2118). Next day, membranes were washed three times with TBST before adding HRP‐labeled secondary antibodies (1:8000, ZSGB‐BIO, ZB‐2301, ZB‐2305). Signals were detected by a chemiluminescent HRP substrate (Millipore) on the Bio‐Rad imaging system (Bio‐Rad ChemiDoc MP).

### 2.6 Experimental Metastasis Model in NOD/SCID Mice

Six‐week‐old NOD/SCID female mice were purchased from Charles River Laboratories (Beijing, China) and maintained under specific pathogen‐free (SPF) conditions. All animal experiments were performed under the guidelines of the Institutional Animal Care and Use Committee at Capital Medical University (Beijing, China) and the Medical Research Center of Beijing Chao‐Yang Hospital. On injection day, 20 mice were randomly divided into two groups, the prepared single‐cell suspensions of MDA‐MB‐231 Tn‐positive cells and control Tn‐negative cells were injected into mice via tail vein (1 × 10^6^/200 μL cells per mouse). All mice were sacrificed 6 weeks postinjection. The lungs were excised and the nodules on the lungs of each mouse were counted. Excised tissues were paraffin‐embedded for hematoxylin and eosin (H&E) staining.

### 2.7 Immunofluorescence Analysis

Cells were cultured on sterile glass coverslips on the day before staining. Cells were fixed in 4% paraformaldehyde, quenched with 50 mmol/L NH_4_Cl, permeabilized in 0.2% Triton X‐100, and blocked in 3% BSA. Subsequently, actin stress fibers were stained with rhodamine‐conjugated phalloidin (Sigma‐Aldrich) for 1 h, and nuclei were counterstained with DAPI. Sections were mounted with Prolong Gold Antifade Reagent. Finally, cell protrusions were visualized using a fluorescence microscope.

### 2.8 Statistical Analysis

Each experiment was independently repeated at least three times. Data analysis was performed using the SPSS 22.0 statistical software. The data from in vitro experiments were presented as the mean ± SD. The differences of survival time between the mice bearing MDA‐MB‐231 Tn‐positive cells and Tn‐negative cells in the metastatic models were evaluated using the log‐rank test. The Student's *t*‐test was used to analyze differences between two groups. Chi‐square test was used to compare immunohistochemistry results and clinicopathological factors. The analysis of the survival curves was performed using the Kaplan–Meier method. A *p*‐value of < 0.05 was considered statistically significant.

## 3 Results

### 3.1 Clinical Significance of Tn Antigen Expression in Breast Cancer Patients

**FIGURE 1:**
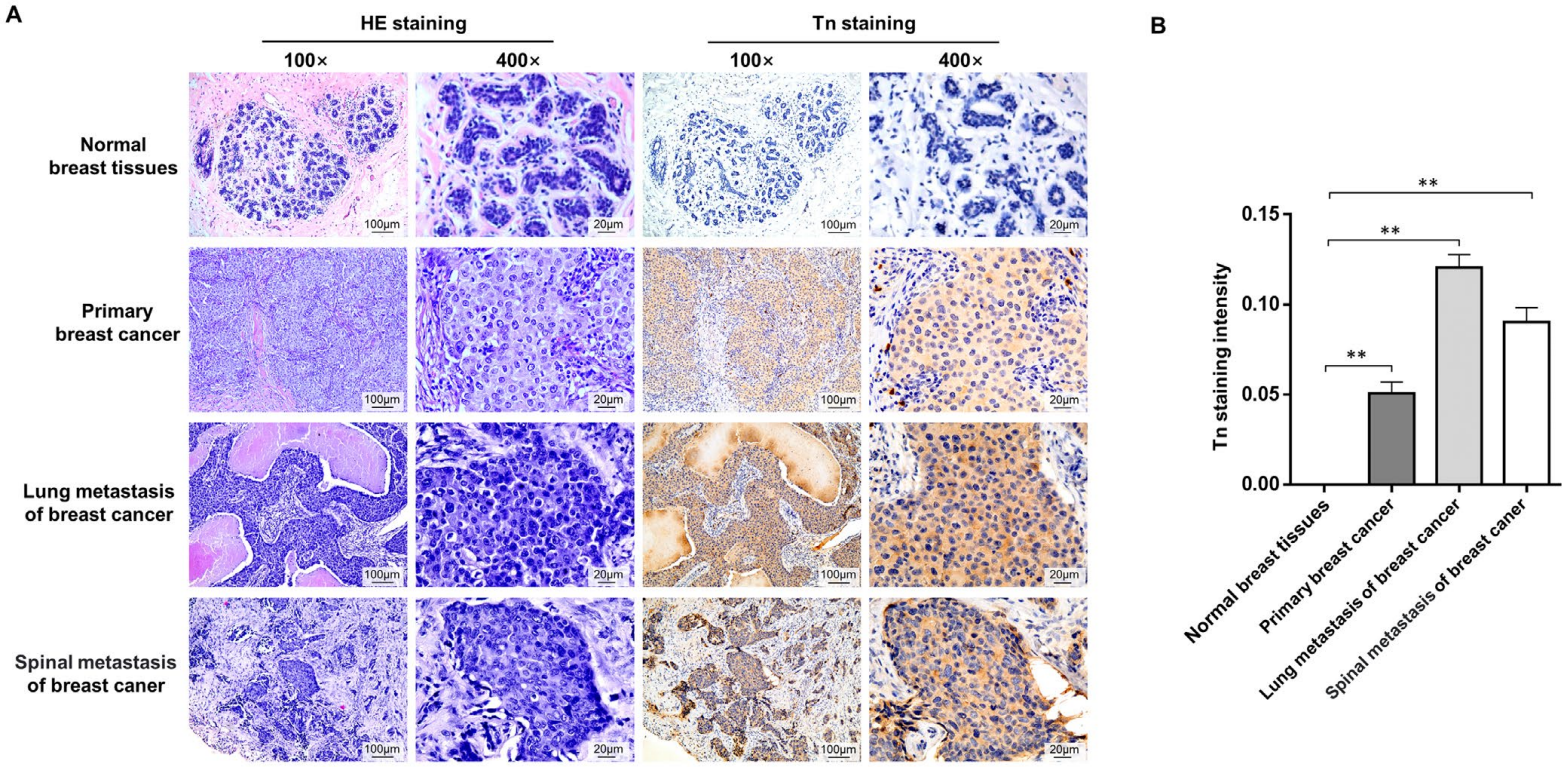
Representative immunohistochemistry staining of Tn antigen in primary breast cancer tissues and distant metastasis tumor tissues. (A) Tn antigen was absent in normal breast tissues, whereas it showed positive expression in primary breast cancer tissues, lung metastasis, and spinal metastasis. All scale bars are 100 μm. (B) Positive staining of Tn antigen was quantified. Error bars indicate the mean ± SD, with significant differences indicated (**p < 0.01).

To assess the clinical significance of Tn antigen in breast cancer, we examined the expression of the Tn antigen in 236 archived human breast tissue samples (12 normal breast mammary epitheliums, 135 primary breast cancer tissues, 39 lymph node metastases, 24 lung metastases, and 26 bone metastases) by IHC analysis. We observed that 208 of the 224 (92.9%) human breast cancer samples were Tn‐positive, whereas there was no Tn antigen staining in normal breast epithelia (0/12). Positive Tn antigen staining was primarily observed in the cytoplasm and/or cell membrane of cancer tissues (Figure 1). Notably, Tn antigen expression was significantly higher in patients with metastatic lesions (97.8%) compared to those with primary lesions (89.6%, *p* = 0.021), suggesting a metastatic‐promoting role for Tn antigen (Table S1).

We further analyzed the association between Tn antigen expression levels and the clinicopathological characteristics of human primary breast cancer (*n* = 135). The levels of Tn antigen immunoreactivity were classified into two categories: “Tn‐low expression” (weak expression, around 0%–35% of cells stained) and “Tn‐high expression” (moderate and strong expression, more than 35% of cells stained) [14, 15]. We found that high Tn antigen expression was significantly correlated with lymph node metastasis (*p* = 0.0013), but not with age, clinical stage, histological type, molecular subtypes (Luminal A, Luminal B, Her2+, or Triple‐negative) (Table S2). We next sought to evaluate the correlation between Tn antigen expression levels and overall survival (OS) in these breast cancer patients. Kaplan–Meier analysis revealed that patients with high Tn antigen expression had a significantly shorter survival rate than those with low Tn antigen expression (*p* = 0.003) (Figure S1). Collectively, these data reveal that Tn antigen is highly associated with cancer metastasis and poor prognosis in breast cancer patients.

### 3.2 Cosmc Depletion‐Induced Tn Antigen Expression Promotes Metastatic Features in Human Breast Cancer Cells

As the clinical relevance of Tn antigen with breast cancer metastasis has been established, we have further investigated the biological effects of Tn antigen expression on metastatic features in cancer cells. We first used the CRISPR/Cas9 technology to knock out the gene encoding Cosmc, which is specifically required for the process of O‐glycosylation (Figure S2A), in two highly metastatic breast cancer cell lines (BT549, MDA‐MB‐231). Disruption of Cosmc was confirmed by western blot analysis (Figure S2B). It showed that T‐synthase, the only enzyme that galactosylates the Tn antigen to form mature core 1‐derived O‐glycans [16], was also absent in Cosmc‐deficient cancer cells, which was in accordant with previous reports that Cosmc is a required molecular chaperone for active T‐synthase formation [17, 18]. Flow cytometry analysis showed that both cell lines deficiency in Cosmc displayed an abundant expression of Tn antigen (Tn‐positive), whereas the mock‐transfected control cells expressed no Tn antigen (Tn‐negative) (Figure S2C).

**FIGURE 2:**
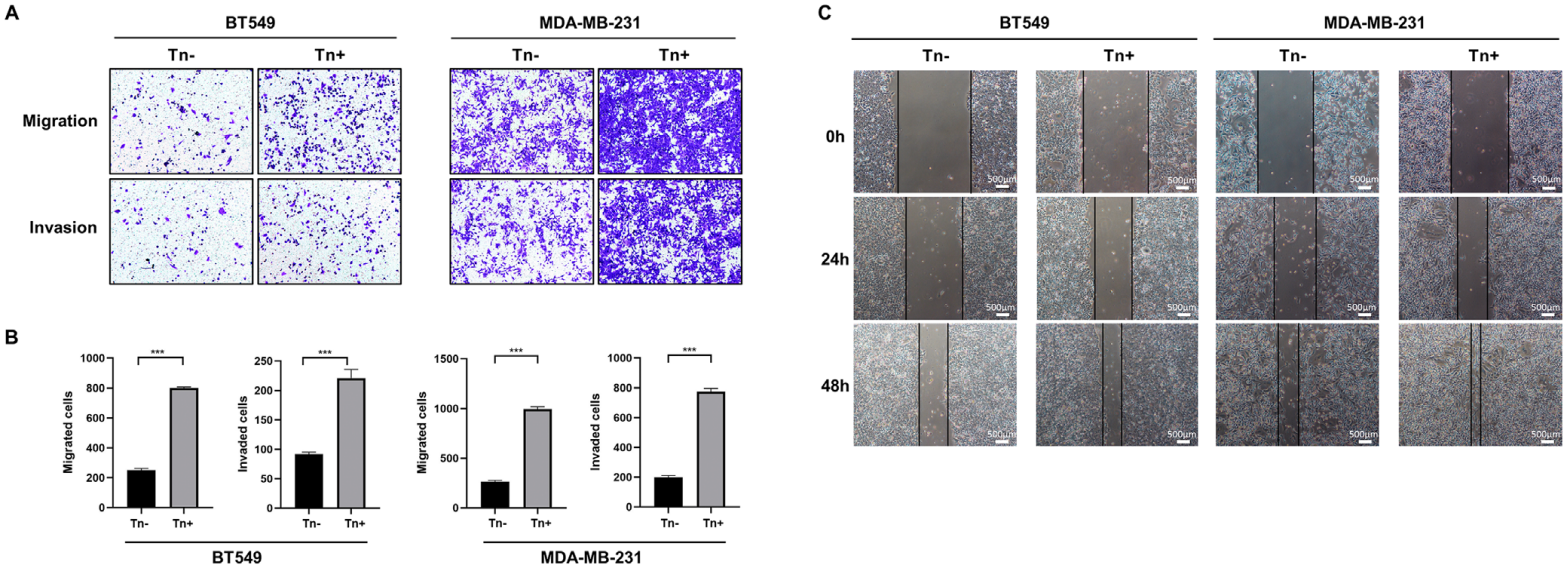
Tn antigen promotes migration and invasion properties. (A, B) The significant increased migration and invasion in Tn‐positive breast cancer cells. The histograms illustrate the quantification of migrating and invading cells, indicating a statistically significant difference (***p < 0.001) (C). The wound healing assays demonstrated a significantly higher motility in Tn‐positive cells compared to Tn‐negative cells.

We then conducted Transwell assays, using Matrigel‐coated inserts for invasion and uncoated inserts for migration, along with a wound‐healing assay to assess the impact of Tn expression on migration and invasiveness in breast cancer cell lines. As shown in Figure 2A,B, both Tn‐positive cell lines displayed enhanced cell migration and invasion compared with the control Tn‐negative cells. In addition, wound healing assay showed that Tn antigen expression remarkably enhanced the migratory speed of BT549 and MDA‐MB‐231 cells compared with that of the vector control cells (Figure 2C). These results supported that Tn antigen expression could boost metastatic capacities of breast cancer cells.

**FIGURE 3:**
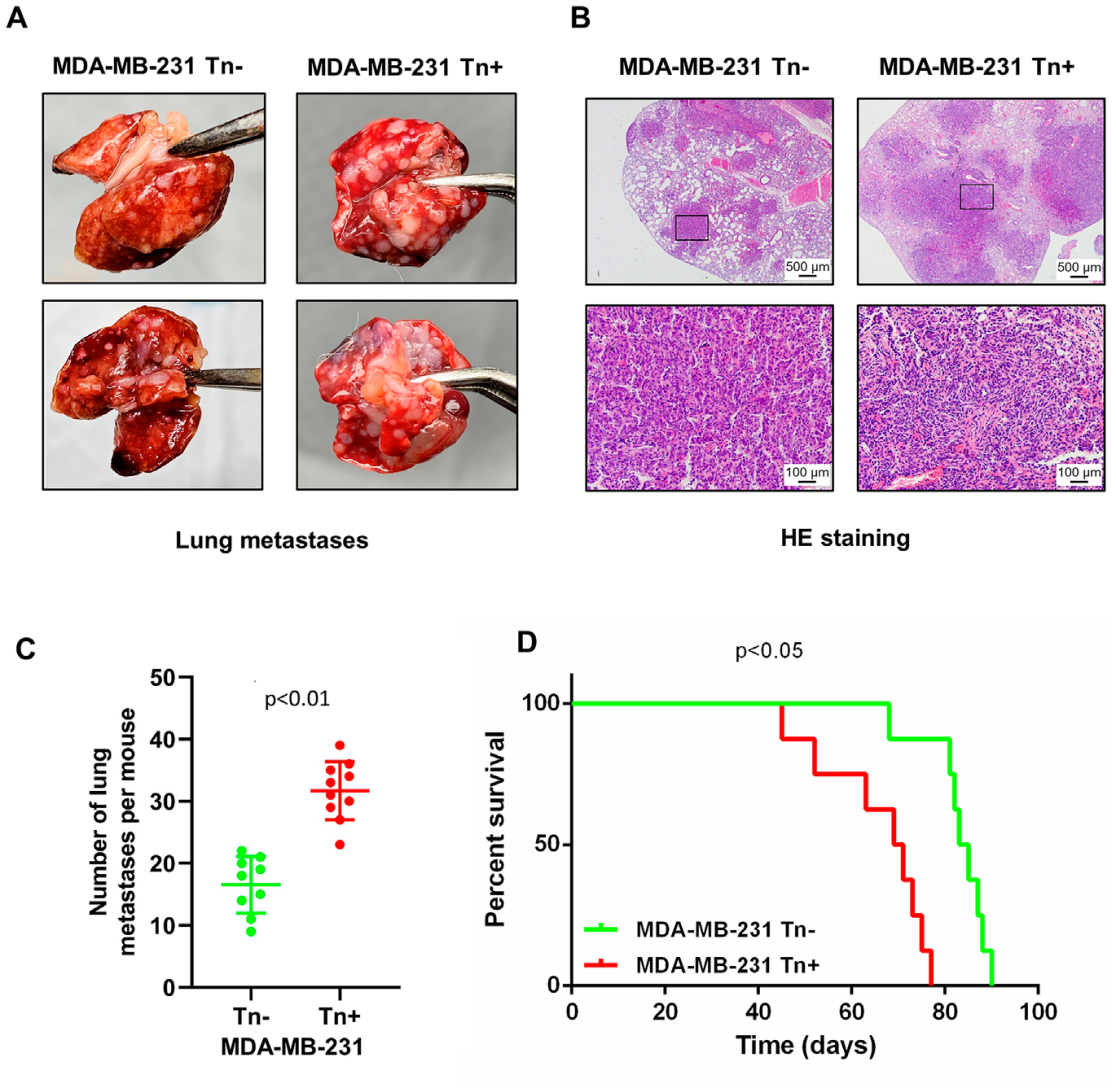
Breast tumors expressing Tn antigen exhibit increased metastatic capacity. (A) Photographs of lung metastases formation in tail vein injection mouse models. (B) Representative HE staining of mouse lung tissue sections (original magnification ×40 and ×200) were shown. (C) Quantities of lung metastases in breast tumor. (D) The analysis of survival rates in mice models with metastatic tumors indicated a notable decrease in survival among those injected with Tn‐positive cells.

Since these in vitro experiments revealed that Tn antigen was associated with prometastatic traits, we next tested whether Tn antigen could accelerate metastasis in vivo using a lung metastatic mouse model. Tn‐positive MDA‐MB‐231 cells and the corresponding control cells (Tn‐negative) were injected into 6‐week‐old NOD‐SCID mice by tail vein. After 6 weeks of inoculation, all mice were sacrificed to evaluate lung metastasis. As shown in Figure 3A,B, the mice injected with Tn‐positive cells developed more lung metastases than those injected with Tn‐negative cells. H&E staining confirmed tumor presence in lung tissues (Figure 3C). Kaplan–Meier survival analysis showed that the mice bearing Tn‐positive xenotransplants had significantly reduced survival times when compared with those with Tn‐negative xenotransplants (*p* = 0.047) (Figure 3D). Collectively, these data indicated that Tn antigen expression could prominently promote tumor metastasis in vivo and result in a worse prognosis.

### 3.3 Tn Antigen Induces Activation of EMT and Cellular Protrusion Formation in Breast Cancer Cells

**FIGURE 4:**
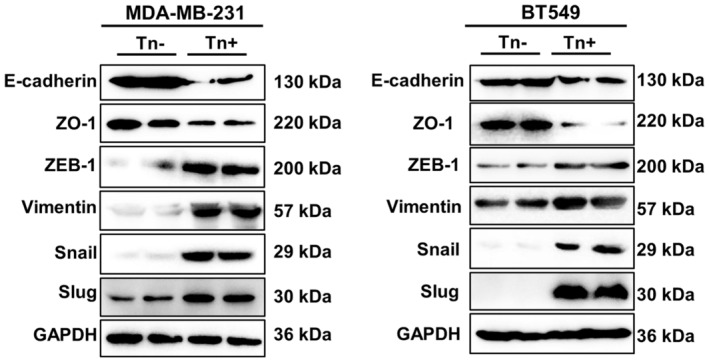
Tn antigen promotes epithelial‐mesenchymal transition in breast cancer cells. Western blot assay revealed a decrease in the expression of epithelial markers, namely E‐cadherin and ZO‐1, along with an increase in the expression of mesenchymal markers, including ZEB‐1, Vimentin, and the transcription factors Snail and Slug. The clear background is caused by the Bio‐Rad imaging system, which helps highlight the detected bands.

Because EMT plays a crucial role in cancer metastasis [19], we have thereby examined whether the EMT program is activated following Tn antigen expression. As shown in Figure 4, both Tn‐positive cell lines exhibited typical EMT characteristics when compared with Tn‐negative control cells. There was a significant downregulation of E‐cadherin and ZO‐1, both of which are canonical epithelial markers, along with a significant up‐regulation of the mesenchymal markers, including ZEB‐1, Vimentin, Snail, and Slug in both cells expressing Tn antigen.

**FIGURE 5:**
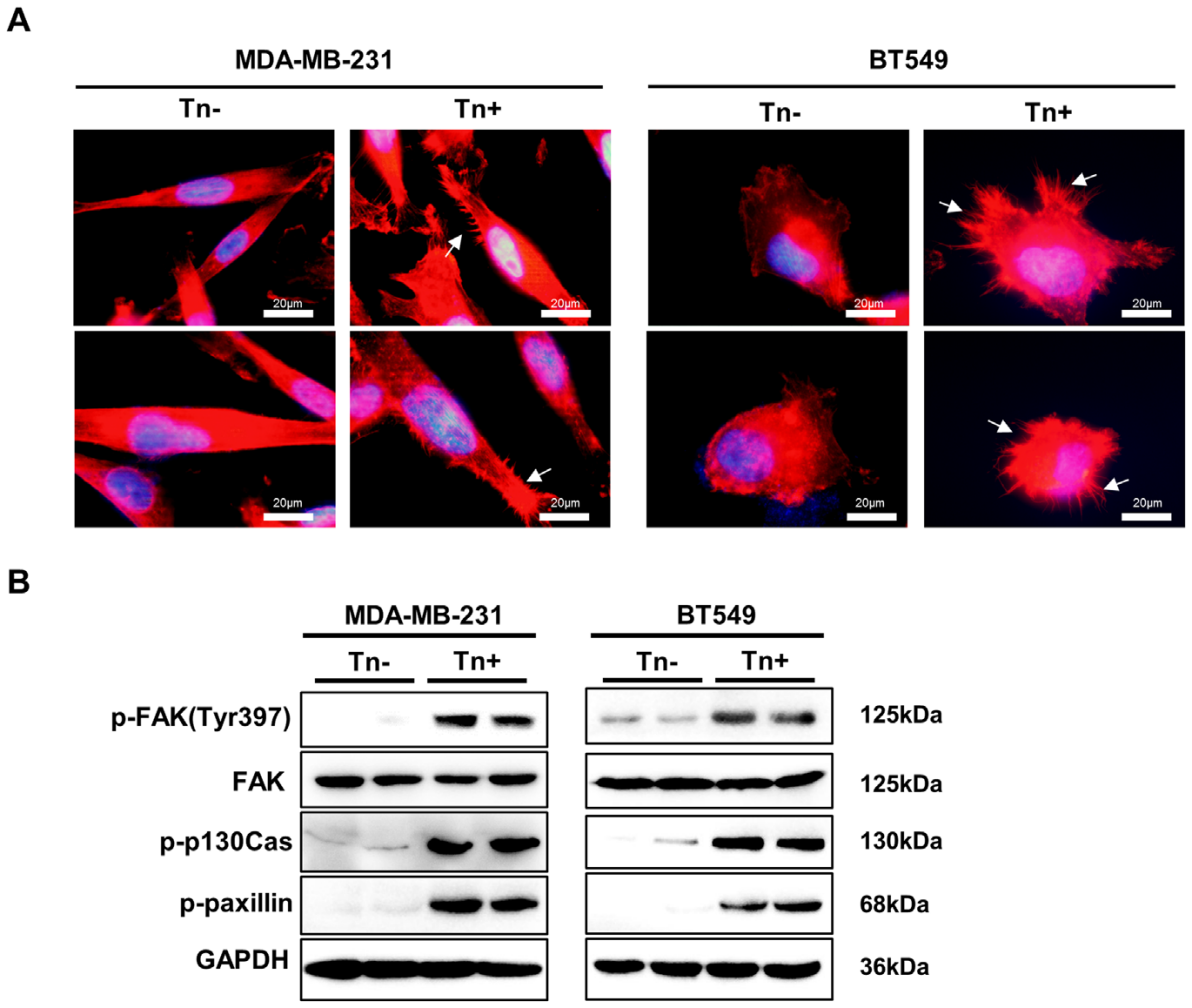
Tn antigen facilitates FAK phosphorylation and the formation of cellular protrusion. (A) The immunofluorescence images of F‐actin staining indicated a significant increase in protrusions formation (white arrow) in Tn‐positive cells. (B) Western blot analysis of FAK, phosphorylated FAK (p‐FAK), phosphorylated paxillin (p‐paxillin), and phosphorylated p130cas (p‐p130cas) were determined. The clear background, is caused by the Bio‐Rad imaging system, which helps highlight the detected bands.

Additionally, immunofluorescence analysis showed that Tn antigen induced altered morphological characteristics, as demonstrated by the increased formation of cellular protrusions, including filopodia and microspikes, both of which are dynamic cytoskeletal structures implicated in cell motility and play an important role in tumor dissemination and metastasis (Figure 5A) [20, 21]. To further delineate the molecular regulators that confer enhanced invasiveness and metastasis in Tn‐positive cells, we have examined the phosphorylation of the FAK signaling cascade including FAK, p130cas and paxillin, in BT549 and MDA‐MB‐231 Tn‐positive cells. It showed that Tn antigen expression significantly increased the phosphorylation levels of FAK (Y397), p130cas and paxillin. Together, these data indicated that Tn antigen expression could promote metastatic features in breast cancer cells via activation of the EMT as well as FAK signaling pathways (Figure 5B).

### 3.4 Blockade of Tn Antigen by HPA Inhibits Metastatic Capacities in Breast Cancer Cells

**FIGURE 6:**
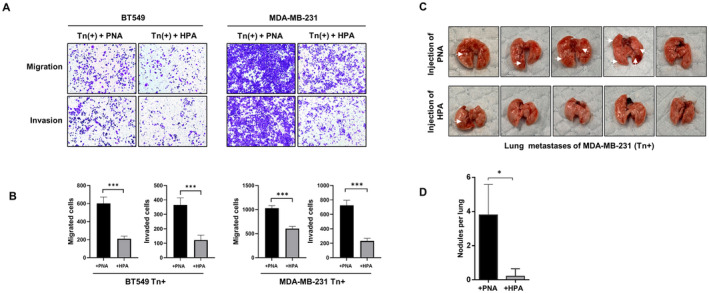
The metastatic capacity of Tn‐positive cells is significantly reduced through the administration of HPA treatment. (A, B) The properties of cell migration and invasion in Tn‐positive cells with HPA and the control lectin PNA were analyzed by Transwell assays. The histograms illustrate the quantification of migrating and invading cells, indicating a statistically significant difference (***p < 0.001). (C) In the NOD/SCID mice lung metastasis model, the combination of tail vein injection of Tn‐positive MDA‐MB‐231 cells with HPA treatment effectively inhibited the in vivo metastatic process, in comparison to the control group treated with PNA. (D) Quantification of lung metastatic nodules in the NOD/SCID mouse model injected with Tn‐positive MDA‐MB‐231 cells, comparing HPA‐treated mice to the PNA‐treated control group (*p < 0.05).

**FIGURE 7:**
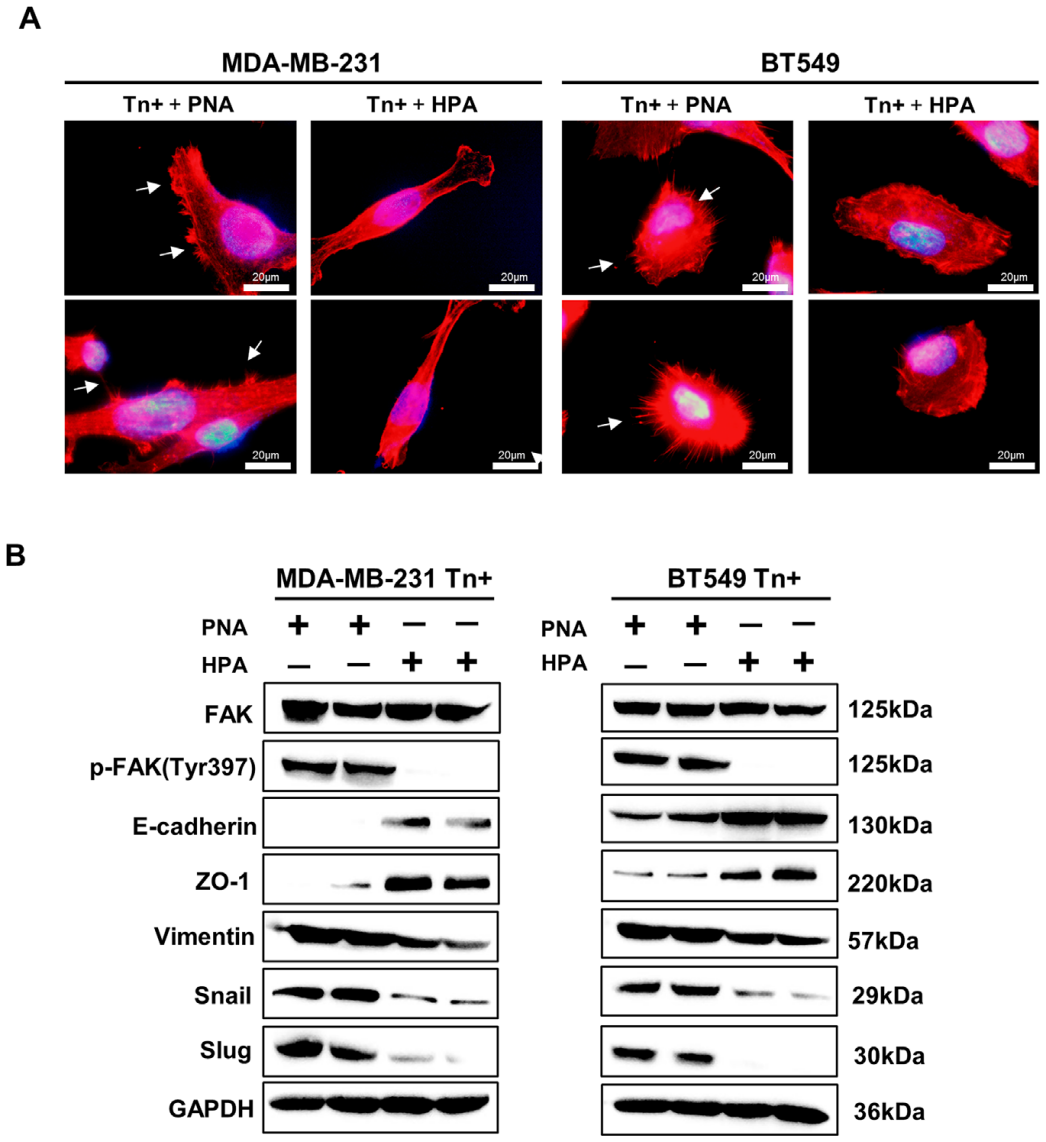
HPA treatment inhibits protrusion formation and EMT activation. (A) The F‐actin staining images revealed the application of HPA treatment resulted in the inhibition of protrusions on the surface of breast cancer cells when compared with the control PNA treatment. (B) Western blot analysis showed the inhibitory effects of HPA on the FAK signaling pathway and the EMT program in contrast to PNA. The clear background, is caused by the Bio‐Rad imaging system, which helps highlight the detected bands.

Since the metastatic‐promoting properties of Tn antigen have been corroborated, we have next investigated whether blockade of Tn antigen has the potential to suppress cancer metastasis. It is known that the lectin HPA (*Helix pomatia agglutinin*) specifically recognizes and binds the Tn antigen, whereas the lectin PNA (*Arachis hypogaea agglutinin*) only recognizes and binds T antigen (core 1 O‐glycans) [22, 23, 24]. Here we treated Tn‐positive cells with HPA to block Tn antigen, whereas PNA was used as the control. It showed that the migration and invasion abilities of both BT549 and MDA‐MB‐231 Tn‐positive cells were significantly decreased in HPA‐treated group in contrast to PNA‐treated group (Figure 6A,B). Furthermore, the HPA‐mediated inhibitory effects were recapitulated in MDA‐MB‐231 Tn‐positive NOD/SCID mouse model. Compared to the PNA‐treated control group, mice in HPA‐treated group exhibited a significantly reduced number of pulmonary metastases (Figure 6C,D). In addition, immunofluorescence analysis showed that HPA treatment reduced formation of cellular protrusions of Tn‐positive cells, whereas PNA showed no inhibitory effects (Figure 7A). At the molecular level, the EMT and FAK signaling pathways were consistently inhibited in both Tn‐positive cells treated with HPA (Figure 7B). All these in vitro and in vivo experiments suggest that targeting Tn antigen has the potential to repress breast cancer metastasis.

## 4 Discussion

Breast cancer metastasis is a significant contributor to mortality and poses a formidable therapeutic challenge in clinical practice [25, 26]. Despite successful resection or chemotherapy in the early stages, distant metastasis remains unmanageable, leading to significantly reduced survival rates among breast cancer patients [27]. Thus far, the key factors governing the complex and sequential processes of metastasis have not been sufficiently elucidated. There is an urgent need to identify potential targets within the regulatory networks for intervention against breast cancer metastasis.

Basically, mucin‐type O‐glycosylation, a post‐translational modification in which carbohydrate moieties are attached to the serine and threonine residues on protein substrates, has been found involved in numerous physiological processes, including cell recognition, adhesion, differentiation, and signal transduction [28, 29, 30]. Accordingly, aberrant O‐glycosylation, which is characterized by the expression of Tn antigen, has been frequently reported in various disease contexts, particularly in human cancers, including those of breast [31], esophagus [32], stomach [33], pancreas [34, 35], colorectum [36, 37], lung [38], prostate [39], thyroid [40], and malignant melanoma [41]. Accumulating evidence suggests a correlation between Tn antigen and cancer progression as well as metastasis. Nevertheless, whether Tn antigen plays a causal and targetable role in breast cancer metastasis remains uncertain.

In the current study, we first investigated clinical relevance of Tn antigen in breast cancer patients through IHC analysis. We observed that Tn antigen was prevalently expressed in human breast cancer, including primary and metastatic lesions, whereas no Tn staining was detected in normal breast tissues. More importantly, we found that the percentage of Tn antigen expression was significantly higher in metastatic lesions than in primary breast cancer samples, and the expression levels of Tn antigen were inversely associated with overall survival in cancer patients. These findings imply that Tn antigen may play a contributing role in breast cancer metastasis. We further explored the mechanisms by which Tn antigen promotes breast cancer metastasis. As Cosmc is the exclusive chaperone for T‐synthase and plays a crucial role in the process of O‐glycosylation, genetic deletion of Cosmc has been commonly used as a research strategy to forcibly induce Tn antigen on cells [34, 42, 43]. In this study, we employed a gene‐editing system to disrupt Cosmc in two highly metastatic breast cancer cell lines to generate Tn‐positive cells. As expected, disruption of Cosmc resulted in the degradation of T‐synthase and abundant expression of the Tn antigen, which resultantly enhanced migration and invasion capabilities of these cancer cells. In addition, nude mice harboring Tn‐positive tumors developed more pulmonary metastatic nodules and had a shorter survival time than the control mice harboring Tn‐negative tumors. Therefore, the results of these functional experiments consistently support a metastasis‐promoting role for Tn antigen.

EMT is known to play a key role in cancer progression to metastasis, as polarized epithelial cells undergo a phenotypic shift, transitioning into nonpolarized mesenchymal cells with heightened mobility [44, 45]. Here, we observed that the EMT program is significantly activated in Tn‐positive cancer cells, which may translate to increased invasiveness and metastasis. Furthermore, EMT activation is often associated with altered cell morphology. We found an elevated formation of cellular protrusions, including filopodia and lamellipodia, in Tn‐positive cells, which may also contribute to cell migration and invasion [46, 47]. We further investigated the molecular mechanisms by which Tn antigen induces the altered morphology. Aberrant activation of the FAK signaling pathway (FAK/paxillin/p130cas axis) is known to drive cancer progression and metastasis, which may promote cell motility by altering the dynamics of the actin cytoskeleton [48]. Here we observed significantly increased phosphorylation of FAK, paxillin, and p130cas in Tn‐positive cells when compared to Tn‐negative cells, which may underlie the enhanced formation of cellular protrusions. Altogether these results suggest that Tn antigen may contribute to breast cancer metastasis by triggering the EMT process as well as the FAK signaling pathway.

Considering that Tn antigen is specifically expressed on malignant cells but not on normal cells and exerts a prometastatic effect, it is likely to become an ideal target for cancer therapy. Lectin possesses the ability to selectively bind a variety of oligosaccharides, rendering it extensively employed for the identification of glycans on tumor cell surfaces [49, 50]. Of these, HPA demonstrates particular specificity for its recognition and binding affinity towards Tn antigen [13, 51]. Here we treated Tn‐positive cells with HPA and found that metastatic capabilities of cancer cells were significantly repressed, along with a reduction in the formation of lung metastases. Targeting Tn antigen also resulted in a considerable decrease in the number of pseudopodia present in Tn‐positive cells, as well as the reversal of the EMT process and the suppression of FAK signaling pathway. The inhibitory effect by HPA is Tn specific, as PNA, which recognises and binds T antigen, showed no effects.

## 5 Conclusions

In conclusion, our work provides novel insights into the molecular mechanisms underlying breast cancer metastasis and facilitates the identification of novel antimetastatic targets. Tn antigen may become a potential antimetastatic target in breast cancer, which is promising but requires further investigations in the future.

## Author Contributions

**Tan Du:** investigation (equal), methodology (equal), writing – original draft (equal). **Xichen Dong:** investigation (equal), methodology (equal), writing – original draft (equal). **Jingyu Tan:** data curation (equal), methodology (equal). **Xiangyu Chen:** data curation (equal), formal analysis (equal), methodology (equal). **Jian Liu:** conceptualization (equal), supervision (equal). **Tao Wen:** conceptualization (equal), funding acquisition (lead), supervision (equal), writing – review and editing (equal). **Xiaoli Ru:** conceptualization (equal), supervision (equal), validation (equal), writing – review and editing (lead).

## Conflicts of Interest

The authors declare no conflicts of interest.

## Supporting information


Data S1.

Figure S1.

Figure S2.

Table S1.

Table S2.

## Data Availability

The datasets used or analyzed during the current study are available from the corresponding author on reasonable request.
