# Supplementary material for: Targeting Tn Antigen Suppresses Aberrant O‐Glycosylation‐Elicited Metastasis in Breast Cancer
Source: J Cell Mol Med. 2024 Dec 9;28(23):e70279. doi: 10.1111/jcmm.70279 (PMC11628356; doi:10.1111/jcmm.70279)
Supplement: Supplementary file 1 — Data S1. [file JCMM-28-e70279-s004.docx]

**Supporting information**

**Methods**

1. **Generation of Cosmc knockout breast cancer cells**

Depletion of Cosmc in breast cancer cells was carried out using the CRISPR/Cas9 gene-editing system. Briefly, The Cosmc-specific guide RNA sequence (F:5′-CACCGTGTGCTTTGATCACTATGCT-3′ R:5′-AAACAGCATAGTGATCAAACACAC-3′) were designed and cloned into LentiCRISPRv2 plasmids, which were co-transfected into HEK293T cells with psPAX2 and pMD2.G plasmids using Lipofectamine 3000 (Invitrogen, Carlsbad, CA, USA) to produce lentivirus containing CRISPR/Cas9 system targeting Cosmc gene. The virus was transfected into MDA-MB-231 and BT549 cells with polybrene (Genechem, Shanghai, China), respectively. These transfected cells were cultured for 48 h and then selected with 2 μg/mL puromycin.

**2. Flow cytometry**

For the analysis of Tn-positive cell population, cultured Cosmc knockout breast cancer cells were collected, washed and suspended in cold PBS. Then the cells (1× 10^6^) were incubated with anti-Tn IgM mAb (10 μg/mL, CA3638, clone 12A8-C7-F5) at 4°C for 1 h followed by incubation with PE-labeled goat anti mouse IgM (Santa Cruz, sc-3768) for 1 h. After washing twice with PBS, the cells were analyzed by flow cytometer (BD bioscience).

**Supplementary Figure legends**

**S Fig 1. Kaplan-Meier analysis for breast cancer patients grouped into high or low Tn expression.**

Patients with Tn-high expression had lower survival rate compared to those with Tn-low expression.

**S Fig 2. Deletion of Cosmc effectively** **elicits the expression of Tn antigen.**

**A.** Picture illustrated the biosynthesis of O-glycan and the key role of Cosmc chaperone in the process of elongation of Tn antigen. **B.** Cosmc and T-synthase were detected in Cosmc-deficient MDA-MB-231 and BT549 by western blot analysis. **C**. Analysis of Tn antigen on cell surface by flow cytometry in Cosmc depletion breast cancer cells.
