# Supplementary figures and images for: Targeting Tn Antigen Suppresses Aberrant O‐Glycosylation‐Elicited Metastasis in Breast Cancer
Source: J Cell Mol Med. 2024 Dec 9;28(23):e70279. doi: 10.1111/jcmm.70279 (PMC11628356; doi:10.1111/jcmm.70279)

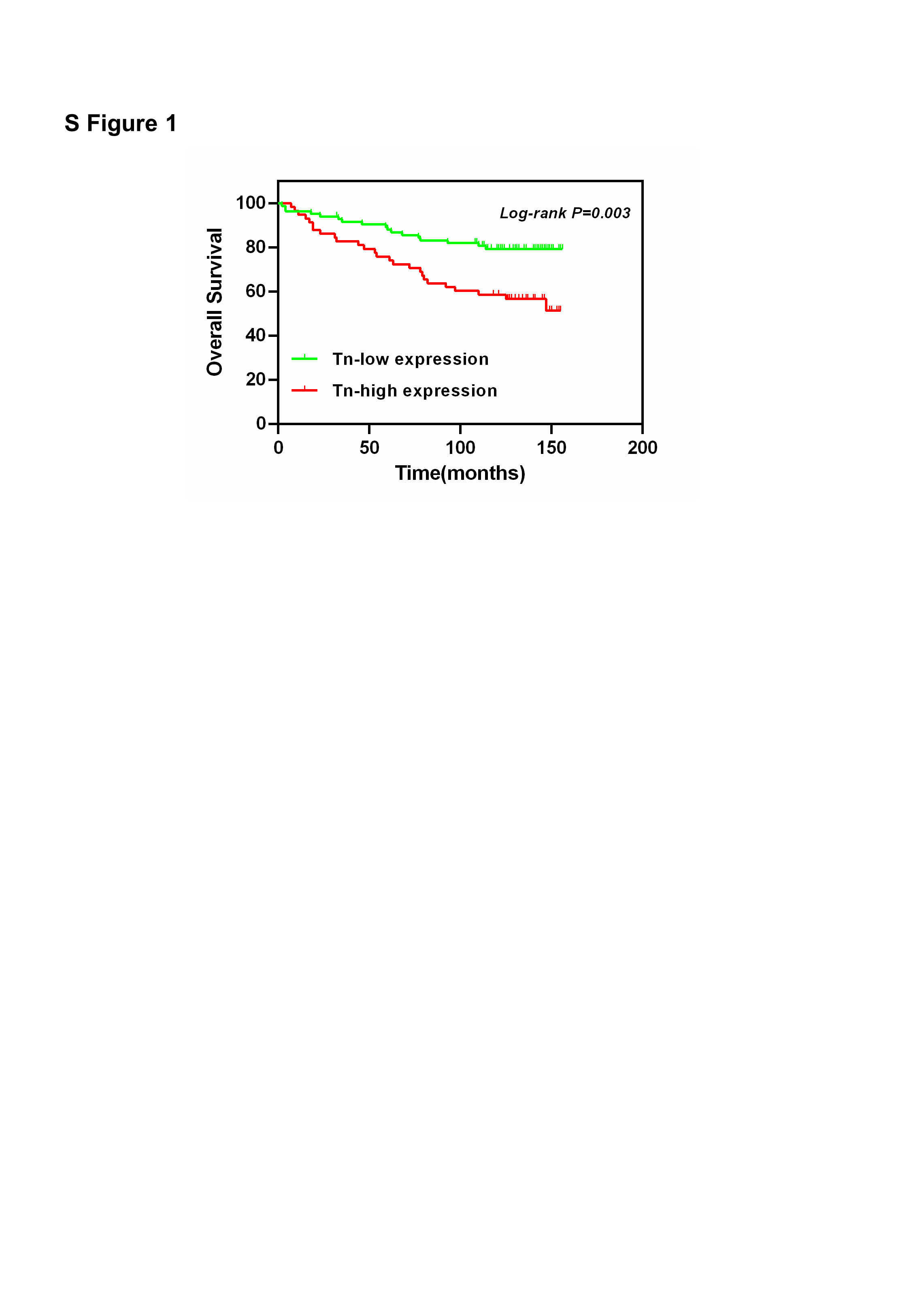

Supplement: Supplementary file 2 — Figure S1. [file JCMM-28-e70279-s005.tif]

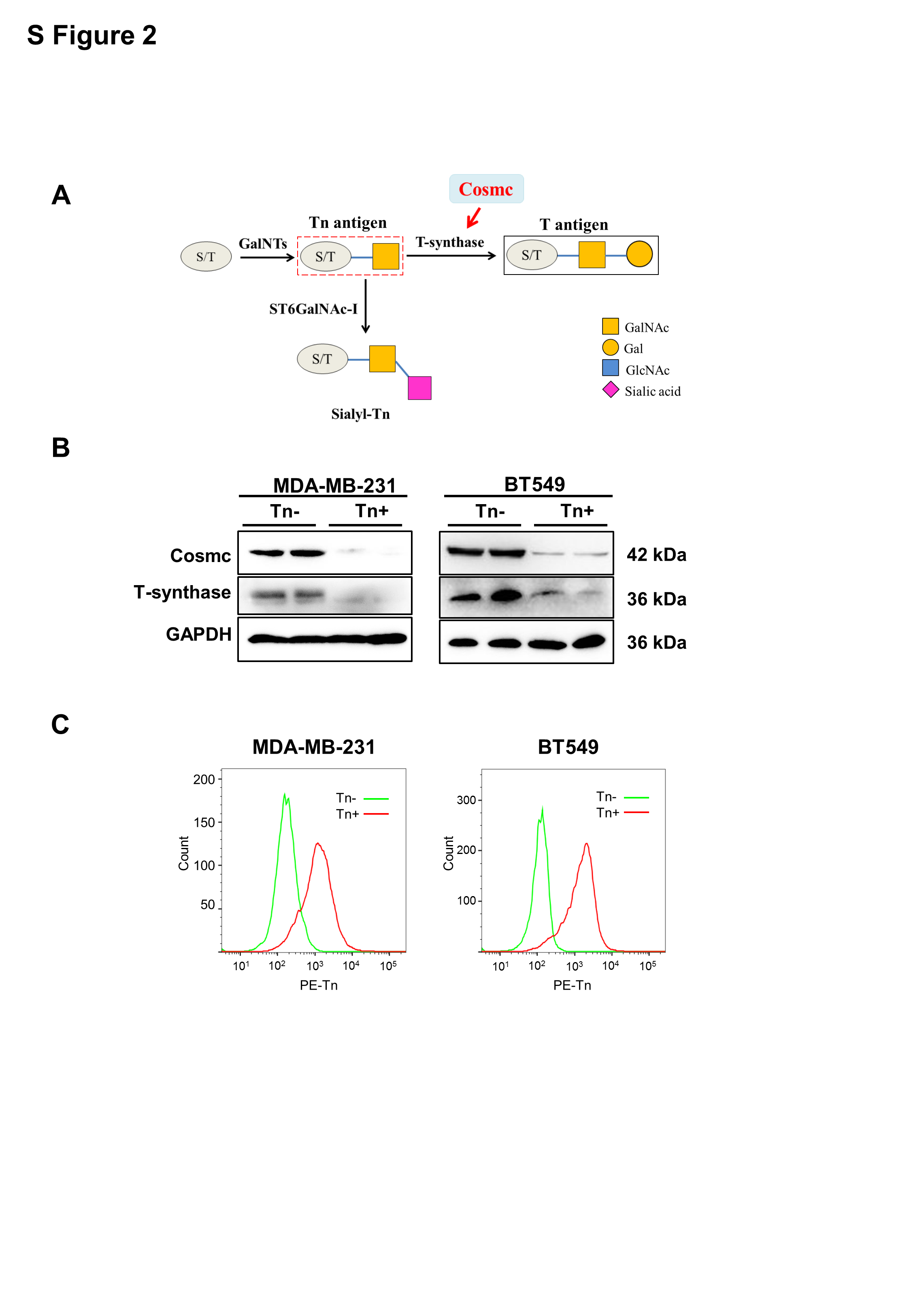

Supplement: Supplementary file 3 — Figure S2. [file JCMM-28-e70279-s001.tif]
