# Supplementary material for: Targeting Tn Antigen Suppresses Aberrant O‐Glycosylation‐Elicited Metastasis in Breast Cancer
Source: J Cell Mol Med. 2024 Dec 9;28(23):e70279. doi: 10.1111/jcmm.70279 (PMC11628356; doi:10.1111/jcmm.70279)
Supplement: Supplementary file 4 — Table S1. [file JCMM-28-e70279-s003.docx]

Supplementary table 1. IHC analysis of Tn antigen in breast cancer patients

| Histological classification | n | Tn antigen expression | | P value |
| --- | --- | --- | --- | --- |
|  |  | Negative | positive |  |
| Normal breast epithelium | 12 | 12 | 0 (0%) |  |
| Primary breast cancer | 135 | 14 | 121(89.6%) | <0.0001 |
| Metastatic lesions | 89 | 2 | 87 (97.8%) | 0.021 |
| Lymph node metastases | 39 | 0 | 39(100%) |  |
| Lung metastases | 24 | 0 | 24(100%) |  |
| Bone metastases | 26 | 2 | 24(92.3%) |  |

The chi-square test was used to analyze the associations between factors. p<0.05 was considered to be statistically significant
