# Supplementary material for: Targeting Tn Antigen Suppresses Aberrant O‐Glycosylation‐Elicited Metastasis in Breast Cancer
Source: J Cell Mol Med. 2024 Dec 9;28(23):e70279. doi: 10.1111/jcmm.70279 (PMC11628356; doi:10.1111/jcmm.70279)
Supplement: Supplementary file 5 — Table S2. [file JCMM-28-e70279-s002.docx]

| Parameters | n | Tn antigen | | P value |
| --- | --- | --- | --- | --- |
|  |  | Low expression | High expression |  |
| Age |  |  |  |  |
| <50 | 58 | 27(46.6%) | 31(53.4%) | 0.563 |
| ≥50 | 77 | 32(41.6%) | 45(58.4%) |  |
| Tumor stage |  |  |  |  |
| Ⅰ | 36 | 21(58.3%) | 15(41.7%) | 0.081 |
| Ⅱ | 40 | 16(40%) | 24(60%) |  |
| Ⅲ | 59 | 20(33.9%) | 39(66.1%) |  |
| Histological type |  |  |  |  |
| Grade Ⅰ | 31 | 20(64.5%) | 11(35.5%) | 0.135 |
| Grade Ⅱ | 46 | 21(45.7%) | 25(54.3%) |  |
| Grade Ⅲ | 58 | 25(43.1%) | 33(56.9%) |  |
| Lymph node metastases |  |  |  |  |
| Absent | 49 | 21(42.9%) | 28(57.1%) | 0.0013 |
| Present | 86 | 15(17.4%) | 71(82.6%) |  |
| Molecular classification |  |  |  |  |
| Luminal A | 67 | 17(25.4%) | 50(74.6%) | 0.2579 |
| Luminal B | 26 | 12(46.2%) | 14(53.8%) |  |
| Her2+ | 18 | 6(33.3%) | 12(66.7%) |  |
| TNBC | 24 | 9(37.5%) | 15(62.5%) |  |

Supplementary table 2. Associations between clinicopathologic characteristics and Tn antigen expression levels in primary breast cancer tissues

The chi-square test was used to analyze the associations between factors. p<0.05 was considered to be statistically significant
